# Removal of the Active Pharmaceutical Substance Entecavir from Water via the Fenton Reaction or Action by the Cyanobacterium *Microcystis novacekii*

**DOI:** 10.3390/toxics12120885

**Published:** 2024-12-05

**Authors:** Cléssius Ribeiro de Souza, Gabriel Souza-Silva, Carolina Paula de Souza Moreira, Olívia Maria S. R. Vasconcelos, Kenia Pedrosa Nunes, Cíntia Aparecida J. Pereira, Marcos Paulo Gomes Mol, Micheline Rosa Silveira

**Affiliations:** 1 Faculdade de Farmácia, Universidade Federal de Minas Gerais, Belo Horizonte 31270-901, MG, Brazil; silva_gs@yahoo.com (G.S.-S.); michelinerosa@gmail.com (M.R.S.); 2 Fundação Ezequiel Dias, Departamento de Pesquisa e Desenvolvimento, Belo Horizonte 30510-010, MG, Brazil; carolina.moreira@funed.mg.gov.br (C.P.d.S.M.); oliviamrv@gmail.com (O.M.S.R.V.); 3 Department of Biomedical Engineering and Science, Florida Institute of Technology, Melbourne, FL 32901, USA; knunes@fit.edu; 4 Instituto de Ciências Biológicas, Universidade Federal de Minas Gerais, Belo Horizonte 31270-901, MG, Brazil; cintiajp@icb.ufmg.br

**Keywords:** entecavir, Fenton-like reaction, degradation, cyanobacteria, *Microcystis*

## Abstract

Entecavir (ETV) is an antiviral used to treat chronic infection caused by the hepatitis B virus, which affects approximately 250 million people worldwide. In order to mitigate the impacts of ETV on the environment, including potential harm to human health, this study evaluated the use of the Fenton-like reaction, which uses iron complexed with ethylenediaminetetraacetic acid (EDTA) at neutral pH, and the microbiological action of *Microcystis novacekii* in removing ETV from the aqueous medium. Aqueous concentrations of 100 mg/L were subjected to Fenton-like degradation. Solutions ranging from 1.2 to 120 mg/L were used for biodegradation. The results evidenced consistent effectiveness in completely removing ETV by the Fenton-like reaction after 90 s. However, removal by the action of *M. novacekii* did not return convincing results. Although entecavir exposure did not affect cyanobacterial cell growth, a gradual reduction in drug content was observed starting on the fourth day of exposure, with maximum removal of 28.9% at the lowest exposure concentration (1.2 mg/L), without, however, showing a significant difference. Statistically significant differences in drug removal were identified only after 14 days of exposure and at specific concentrations. The ETV degradation process through the Fenton reaction was effective and promising for practical application. Removal through *M. novacekii* showed limited efficacy for practical application for its direct use in the remediation of ETV in aquatic environments. However, we identified a slight decrease in the initial concentrations that could achieve greater efficiency in the drug’s degradation through associations with other microorganisms, physiochemical processes, or even genetic engineering.

## 1. Introduction

Entecavir (ETV) is an antiviral medication for chronic hepatitis B virus (HBV) infection [1,2]. According to the World Health Organization (WHO), 254 million people were living with hepatitis B worldwide in 2022, with 1.2 million new yearly infections [3]. A total of 276,646 hepatitis B cases were reported in Brazil from 2000 to 2022 [4].

ETV is a 2-deoxyguanosine analog antiviral with selective activity against hepatitis B virus (HBV) DNA polymerase and acts as a nucleoside reverse transcriptase inhibitor (NRTI). Its principal antiviral activity occurs after intracellular phosphorylation, generating an active metabolite in the form of triphosphate that inhibits all stages of enzymatic activities for viral replication, selectively inhibiting the initiation of HBV DNA polymerase, the reverse transcription of negative-strand DNA of messenger RNA, and the synthesis of positive-strand DNA. The recommended doses are 0.5 mg/day to 1.0 mg/day for compensated and decompensated liver disease, respectively. Approximately 68 to 73% of the drug is eliminated unchanged by the kidneys, and renal clearance is regardless of dose, suggesting that ETV undergoes both glomerular filtration and tubular secretion [5]. The pKa and Log K_ow_ values in Table 1 suggest that ETV persists in the environment in a non-ionized form that is hard to absorb by the lipid layers of the compartments [6]. The detection of entecavir in environmental matrices, including natural waters, was not found in the literature [7].

An exponential increase in new drugs and volumes produced worldwide has been observed with growing scientific and technological development [8,9]. Persistent organic pollutants (POPs) are organic compounds resistant to environmental degradation through biological, chemical, and photolytic processes generating toxicity [10]. Waste from production processes in the chemical and pharmaceutical industries, improperly discarded medicines, and their metabolites associated with consumption are examples of POPs [10,11,12].

Advanced oxidative processes (AOPs) have stood out as an additional alternative to conventional biological effluent treatment processes that mostly use upflow anaerobic sludge blanket reactor (UASB), activated sludge systems, or aerated lagoon systems, with the latter requiring large extensions and considerable time for the degradation of compounds by microorganism action [13,14]. The Fenton reaction and the photo-Fenton process promote the chemical degradation of several substances through the generation and action of hydroxyl radicals (HO•). This radical has an elevated oxidation potential (+2.8 V), higher than that of conventional oxidants and lower only than fluorine’s oxidation potential (+3.03 V) [15].

Other than the traditional Fenton process, adding EDTA to the system oxidizes the compounds at neutral pH, leading to more efficient consumption of H_2_O_2_, more significant decomposition of H_2_O_2_ catalyzed by iron into hydroxyl radicals (HO•), and, consequently, better removal of organic compounds from the sample [16]. Furthermore, due to the neutral pH, additional biological processes can be used for the final degradability of substances [17,18].

Another alternative for effluent treatment is methods that use cyanobacteria, which have great potential for biodegradation systems in ecotoxicological studies [19]. These microorganisms are widely distributed and have well-developed mechanisms of adaptation to environmental conditions, which gives them great resistance [20].

Cyanobacteria and microalgae are essential in reducing environmental organic pollutants through bioaccumulation, biodegradation, removal, or other mechanisms [19,21]. These microorganisms occupy the base of the food chain and can degrade pollutants present in the environment [19,22], including drugs, such as in the biodegradation or removal of carbamazepine [23,24], tetracycline [25], chlortetracycline and oxytetracycline [26], insecticide pyridafenthion [27], and pharmaceutical effluents [21,28].

Due to its importance in ecosystems and its capacity for bioaccumulation and degradation of pollutants, besides being an essential indicator of pollution in aquatic environments, *Microcystis novacekii* can be used to consistently assess pollution levels of different substances and structured use in effluent treatment plants [29,30,31]. Studies show the ability of cyanobacteria of the genus *Microcystis* sp. to degrade drugs and pesticides in the aquatic environment without generating active or toxic metabolites [29,30,32,33].

Considering the possible presence of entecavir in the aquatic environment, an antiviral that directly interferes with enzymatic processes and protein synthesis, with possible impacts on the environment and human health, and the significant excretion rate of the drug in its unchanged form, this research aimed to evaluate the chemical degradation of entecavir by the advanced oxidative Fenton process and the microbiological removal by the action of the cyanobacterium *Microcystis novacekii*.

## 2. Materials and Methods

### 2.1. Supplies and Equipment for ETV Quantification

The Ezequiel Dias Foundation (FUNED) analyzed ETV with 99.88% purity, meeting all the quality requirements specified by the American Pharmacopoeia. The solutions for the chemical degradation tests were prepared with highly pure Milli-Q water at an initial concentration of 100 mg/L. Concentration was monitored by high-performance liquid chromatography (HPLC) in a Shimadzu chromatograph, model LC-2060C 3D, manufactured by Zhimadzu Corporation, Kyoto city, Japan, using a Discovery^®^ HS C18, reversed-phase column (250 × 4.6 mm; 5 µm), manufacture by Merck, Darmstadt city, Germany, flow rate of 1.0 mL/min, scanning from 190 to 800 nm, temperature of 30 °C, and injection volume of 10 µL. Elution was performed with 20% HPLC-grade acetonitrile in water, both acidified with 0.1% formic acid for 10 min. A calibration curve was prepared with the primary ETV standard provided by the European Pharmacopoeia for quantification, and the data were processed using the LabSolutions software, version 5.110, installed on the equipment. Other substances such as H_2_O_2_ 30v, FeSO_4_•7H_2_O, and Na_2_-EDTA for reaction development were purchased with a degree of purity suitable for analysis.

### 2.2. Degradation with Fenton’s Reagent

The 1 mol/L Fe-EDTA complex solution was previously prepared in an aqueous medium with pH close to neutrality. The Fenton reaction was performed in a 250 mL glass beaker under constant magnetic stirring at room temperature and with pH adjusted to 7.0 ± 0.5 with NaOH 0.1 mol/L. In a 100 mL aliquot of the 100 mg/L ETV solution, 10 mL of 1 mol/L Fe-EDTA solution was added to the beaker, followed by 3.8 mL of 30 V H_2_O_2_ to start the reaction. Samples of 1 mL in volume were collected at 0, 5, 40, 90, 180, 300, 600, 900, 1200, 1500, 1800, 3600, 5400, and 7200 s, individually neutralized with 8.7 mg of Na_2_SO_3_. The test was performed in triplicate.

In the presence of organic matter, reactions with Fenton’s reagent can occur through a chain reaction mechanism in which the limiting step is the formation of hydroxyl radicals, in this case, the consumption of H_2_O_2_, as per Equations (1)–(6) [34,35].

(1) $$Fe^{2+}+H_{2}O_{2\;}⇄\;Fe^{3+}+HO^{•}+OH^{-}\;$$

(2) $$R-H+HO^{•}\;⇄\;R^{•}+H_{2\;}O$$

(3) $$R^{•}+H_{2}O_{2\;}\to \;ROH+HO^{•}$$

(4) $$R^{•}+Fe^{3+}\to \;R^{+}+Fe^{2+}$$

(5) $$R^{•}+HO^{•}\to \;ROH$$

(6) $$R^{•}+R^{•}\to \;products$$

### 2.3. Removal with Microcystis novacekii

A cyanobacterium strain, *Microcystis novacekii*, was isolated from water samples collected in Dom Helvécio Lake, in the Rio Doce State Park (42°35′595″; 19°46′419″; Minas Gerais, southeastern Brazil) in May 2004. The non-axenic *M. novacekii* strain is kept in culture in the algae and cyanobacteria bank of the Laboratory of Limnology, Ecotoxicology and Aquatic Ecology at the Institute of Biological Sciences of the Federal University of Minas Gerais (LIMNEA-ICB-UFMG). The study used ETV solutions whose concentrations did not show toxicity to cyanobacteria in an ecotoxicological test previously conducted per OECD guide 201 (Supplementary Materials). The ETV solutions for the removal tests were prepared in an ASM-1 culture medium [36] at the Public Health/Water Laboratory of the Faculty of Pharmacy of the Federal University of Minas Gerais (LASPA-FAFAR). A 240 mg/L ETV stock solution was prepared in an artificial seawater medium 1 (ASM-1). The drug was added to the culture medium under constant stirring until complete solubilization for preparation. Specific volumes of the ETV stock solution and the ASM-1 culture medium were added to Erlenmeyer flasks to a total volume of 100 mL, reaching concentrations of 120, 60, 24, 12, and 1.2 mg/L. The samples were incubated under agitation at a controlled temperature of 23 ± 2 °C, with a 12 h photoperiod, and sampling was performed at 0, 4, 7, 14, 21, and 30 days of incubation. Cell growth was evaluated by visible spectrophotometry at 680 nm after each sampling using mathematical relation 7. A Merck Spectroquant^®^ Prove 100 spectrophotometer, manufacture by Merck, Darmstadt city, Germany was used. The growth curve for the strain, described in mathematical relation 7, was previously established by LASPA-FAFAR. The samples were filtered through a 0.22 µm cellulose ester membrane and sent for ETV quantification. ETV quantification was performed as described in the previous item by HPLC. The test was performed in triplicate, and the process efficiency was determined by mathematical relation 8.

(7) $$Y=10^{7\;}x-10^{6}\;\left(r^{2}=0.9963\right)$$

where *Y* is the number of cells in the medium, and *x* is the absorbance determined in the sample.

(8) $$E=\frac{\left(C_{i}-C_{f}\right)}{C_{i}}\cdot 100$$

where *E* is the process efficiency in percentage, *C_i_* is the initial concentration, and *C_f_* is the final concentration of ETV in the sample.

### 2.4. Statistical Analysis

Statistical analyses were performed by R software, version 4.2.2, using the Shapiro–Wilk tests to assess data distribution, the nonparametric Wilcoxon test, the Kruskal–Wallis test, and the Games–Howell nonparametric post hoc test [37,38].

## 3. Results and Discussion

### 3.1. Degradation by Fenton Reaction

ETV underwent rapid degradation shortly after the start of the Fenton-like reaction. The results showed that ETV was no longer detected (Table 2) after 90 s. No significant pH changes were observed, remaining between 6.8 and 7.2, close to the neutral value.

**Figure 1:**
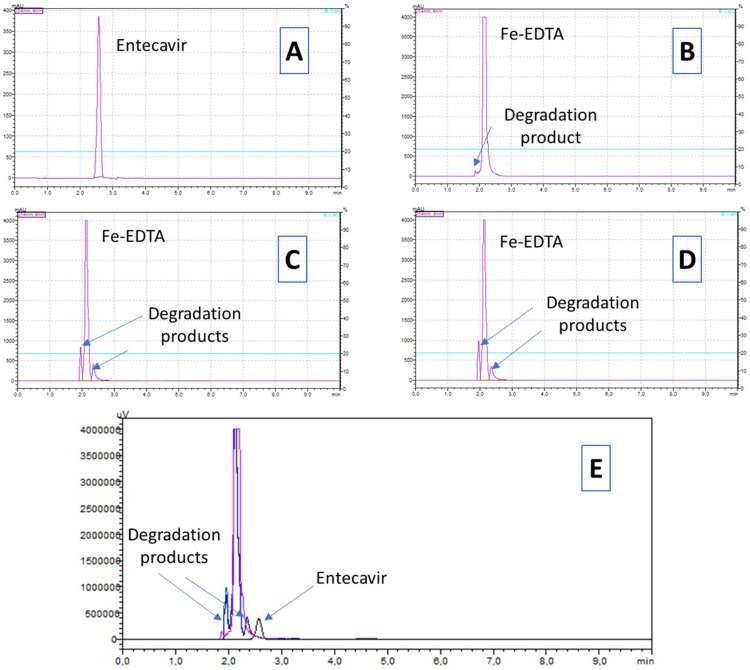
Chromatograms of entecavir degradation using a Fenton-like reagent at different reaction times. Legend: Visualization of entecavir removal over time. Reaction time—(**A**) initial (time zero—no addition of Fe-EDTA); (**B**) 90 s; (**C**) 300 s; (**D**) 600 s; (**E**) chromatogram overlay.

We observed 100% efficiency after 90 s of exposure to the Fenton-like reagent, which is a promising result from a practical viewpoint. However, we could not affirm that ETV was completely mineralized since other peaks were detected in the chromatogram, indicating the occurrence of byproducts (Figure 1). Although EDTA is a complex molecule to degrade due to the presence of EDTA in the matrix, some of the byproducts identified in the chromatogram could stem from both ETV and EDTA. Fenton degradation studies of ETDA show slight-to-moderate reductions at neutral pH and more effective reductions at acidic pH and in the presence of ultraviolet light [16,39,40,41].

ETV degradation is consistent with some studies using the Fenton or Fenton-like reaction with iron complexed with carboxylic acid. A study using effluent from a sewage treatment plant subjected to the Fenton degradation process for 3600 s (60 min) identified several substances with pharmacological activity.

The results were also significant. The initial concentrations of the drugs in the effluent, namely citalopram, at 93 ng/L; codeine, at 37 ng/L; tramadol, at 853 ng/L; and venlafaxine, at 371 ng/L, decreased to concentrations of <4.4 ng/L, <5.5 ng/L, <3.7 ng/L, and <3.7 ng/L, respectively, after the Fenton-like reaction, indicating a removal rate of 90 to 99% of the medicines from the effluent and 76 to 99% of the illicit drugs [42]. Another study showed that dipyrone, an anti-inflammatory widely used in Latin America, achieved removal rates of 94.1% in 45 min [43]. Regarding the specific use of the Fe-EDTA complex, promising results have been achieved for phenol degradation [16]. After 30 min of reaction, 85% of the substance was converted to short-chain organic acids, and 96% was converted to the same acids after 60 min. In another study using the Fe-EDTA complex, 100% degradation of the organic dye malachite green, an aromatic, polycyclic, highly toxic substance used in the cellulose and textile industries and as an antimicrobial in fish farming, was achieved in 90 min [44].

The concentration used for ETV was significantly lower than those studied for malachite green and phenol degradation. In general, the aim is to evaluate degradation processes that meet environmentally relevant concentrations, either due to their actual occurrence in effluents and water bodies or due to the criteria established by the Globally Harmonized System of Classification and Labeling of Chemicals (GHS), which established samples with EC_50_ above 100 mg/L as non-toxic to aquatic ecosystems [45]. Therefore, the differences in the time required for the significant degradation of substances ETV (90 s), phenol (3600 s), and malachite green (5400 s) are directly related to the initial concentrations subjected to the Fenton reagent and, possibly, the respective molecular structures.

All these results converge to the crucial point of using the Fe-EDTA complex, the advantage of using lower concentrations due to the regenerated Fe^2+^ and Fe^3+^, the possibility of total substance mineralization, the execution of the process at a pH close to neutrality, and the lower precipitation rate of Fe^2+^, which, on a real scale, would reduce the operating costs and disposal of the treated effluent into the environment. However, the drawback of the process is EDTA being released into the environment and hydroxyl radicals reacting with EDTA, thus destabilizing the complex and reducing the reaction speed [16,46]. Despite the possible release of EDTA into the environment, there is no consensus on its environmental toxicity, although most authors point to its low toxicity. Currently, EDTA is widely used in the pharmaceutical, cosmetic, and agricultural industries and for the remediation of metal contaminants in the environment [44,47,48,49].

Even with such different results, the processes proposed for removing ETV from aqueous media have advantages and disadvantages. The Fenton-like process has the advantage of mineralizing pollutants or degrading recalcitrant molecules such as drugs, low chemical consumption compared to conventional Fenton treatment, low sludge production, speed, and a considerable reduction in chemical oxygen demand. However, it has the disadvantage of being economically unviable for small and medium-sized industries, allowing for the formation of byproducts that can be more toxic and a low yield if operated outside the ideal conditions, especially considering pH and H_2_O_2_ concentration, and contributing to eutrophication due to the possible degradation of EDTA [13,50].

### 3.2. Microbiological Removal by the Action of the Cyanobacterium Microcystis novacekii

**Figure 2:**
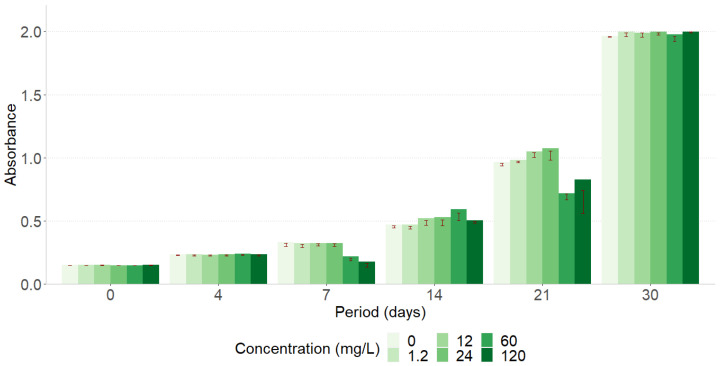
Growth curve of *Microcystis novacekii* exposed to entecavir at different concentrations.

Chronic exposure of *M. novacekii* to ETV (Figure 2) did not affect its cell growth against the negative control. Even in the periods of 7 and 21 days, where a decrease in cell growth was observed at some concentrations, the culture recovered its reproductive capacity in the following period. There were no significant differences for all the other periods evaluated (*p* > 0.05). These results suggest that ETV was not used as a substrate that could provide nutrients capable of promoting differentiated cell growth and that there was no interference in the growth rate; that is, there was no toxicity to the organism.

**Figure 3:**
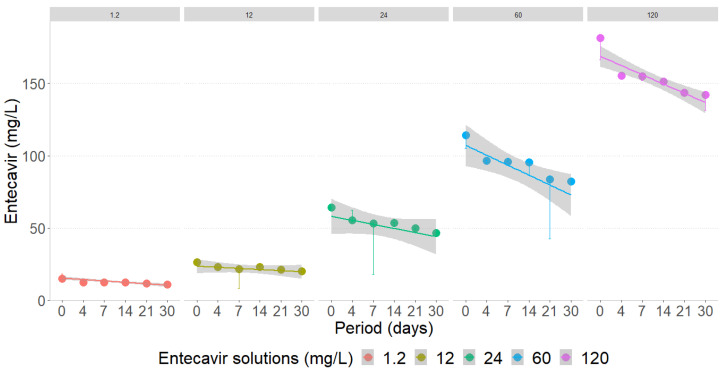
Removal of entecavir at different concentrations by the action of *Microcystis novacekii*, expressed by medians and standard errors.

All ETV concentrations subjected to the action of *M. novacekii* evidenced a slight decrease in the drug content from the fourth day of exposure, with maximum removal of 28.9% at the lowest exposure concentration (1.2 mg/L), however, this difference was not statistically significant (Figure 3; Table 3).

Considering the initial concentration of each exposure block, statistically significant differences in drug removal were observed only after 14 days of exposure for the 12 and 24 mg/L concentrations, but with no practical applicability for effluent or water treatment. In practice, the hydraulic retention time, i.e., the mean time that the hydraulic mass remains inside a tank, should range from 5 to 10 days for the aerobic treatment of sewage and water [51]. Biodegradation or removal depends on the species and characteristics of the xenobiotic molecule, such as liposolubility, transport across the cell membrane, and cell growth. For example, in four days, *M. novacekii* cultures removed 26.06% of atrazine (50 µg/L) and 73.18% α-Ethinylestradiol (0.15 mg/L), and after five days, 58.7% of methyl parathion (1 mg/L) [25,30,32].

Cyanobacterial colonies are characterized by the ability to consume inorganic carbon, causing the pH of surface waters to rise to alkaline levels (close to 9) [52,53]. In general, xenobiotics are found in eutrophic environmental matrices with nitrogen and phosphorus. For *Microcystis* spp., enrichment of the medium with N promotes larger colonies and lower P. Under these conditions, P is not considered a growth promoter of *Microcystis* spp. colonies [52]. The ETV molecule has 5 N atoms that could be degraded to ammonia (NH_3_), which would further corroborate the increase in pH, with consequent ETV hydrolysis in the medium [54,55]. This pH change could promote a competitive advantage for the organism, favoring its growth [52]. The pH increase was not observed during the exposure period, ranging from 5.5 to 7, suggesting that the carbon in the ETV molecule was not completely metabolized to inorganic forms, nor was the nitrogen completely metabolized to ammonia. The sharp change in pH could be attributed to ethylenediaminetetraacetic acid (EDTA) in the culture medium, which could act as a buffering agent. Another possibility would be mixotrophic cell growth, thus reducing the consumption of inorganic carbon [56].

The low removal rates may be related to ETV hydrophilic characteristics (LogK_OW_ = −0.8). Studies by Bai and Acharya (2016) showed that the hydrophobic molecule such as triclosan (LogK_OW_ = 4.76) was better degraded by the green algae *Nannochloropsis* sp. when compared to the molecules of sulfamethoxazole (LogK_OW_ = 0.89) and trimethoprim (LogK_OW_ = 0.91), reaching 100% removal after seven days of exposure [57]. Also, considering the possible biodegradation mechanisms that involve adsorption, absorption, metabolization, and conjugation, more liposoluble (hydrophobic) xenobiotics would be more easily transported into cyanobacteria, facilitating the biodegradation process by enzymatic actions [58].

Cyanobacteria can effectively respond to several organic pollutants, including pharmaceuticals, using bioaccumulation and biodegradation mechanisms [25,56]. Molecules more complex than ETV, such as amoxicillin, sulfamethoxazole, tetracycline, tenofovir, carbamazepine, and malathion, were efficiently removed from the medium by the action of cyanobacteria, including *M. novacekii* [24,25,59,60].

Bioremediation using pure cultures of microorganisms or mixed cultures has the advantage of being simple, economically viable, and widely applied in the market. In addition, they efficiently eliminating organic pollutants and some inorganic pollutants such as ammonia and iron, with a high capacity for removing biochemical oxygen demand and suspended solid material, and it is promising as a new technology for removing emerging contaminants. Its disadvantages include constant monitoring to maintain favorable conditions for the development of microorganisms; the need for pretreatment to reduce the toxicity of substances; a slow removal process; low biodegradability for some molecules such as ETV, dyes, and inorganic substances; the generation of biological sludge and uncontrolled degradation products; requiring efficient management of these byproducts; and the possibility of altering the crops present [50].

The association of molecules with a low percentage of removal from the medium with other organisms can facilitate their degradability. As shown in other studies, recalcitrant and toxic substances were efficiently removed using an association of organisms such as bacteria, algae, cyanobacteria, and macrophytes [61,62,63,64]. This option could be the path for the ETV bioremediation process with *M. novacekii*. However, the potential for degradation of the cyanobacterium *M. novacekii* in uncontrolled environments may pose risks, such as the production of toxins that can significantly harm surface water bodies, especially microcystins. Thus, using a consortium of organisms, mainly other bacteria with the capacity to degrade these molecules, could control or reduce the concentration of microcystins in the aquatic environment [65].

## 4. Conclusions

The Fenton-like reaction demonstrated rapid and efficient ETV removal, making it a promising method for environmental remediation. The 1.5 min exposure time reduced the ETV concentration from 100 mg/L to undetectable levels. ETV may not have been fully mineralized, generating byproducts whose toxicity is unknown. However, ETV molecule de-structuring mitigates the risks of developing resistance to the antiviral in microorganisms and viruses.

Removal by *M. novacekii* was limited for its direct use in the remediation of ETV in aquatic environments, although we observed a slight decline in relation to the initial concentrations submitted to the organism. The lack of data on ETV concentrations in the environment and the results obtained in this study, especially regarding biodegradation or removal, raises a concern about the possible accumulation of this antiviral in the environment with a real possibility of developing resistant strains. Future research should explore synergistic approaches combining biological and chemical processes, with a focus on environmentally relevant ETV concentrations and matrix complexities. Addressing the persistence of pharmaceuticals like ETV is essential for mitigating their ecological and public health impacts.

## Figures and Tables

**Table 1:** Identification and physicochemical properties of entecavir.

Description	Result
CAS No	142217-69-4
Molecular structure	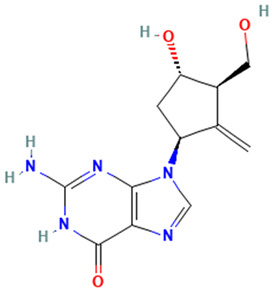
Molecular weight	277.28 g/mol
Chemical formula	C_12_H_15_N_5_O_3_
pKa	8.0
LogK_ow_	−0.8

**Table 2:** The efficiency of entecavir removal by Fenton-like reaction from 0 to 10 min.

Time (s)	Concentration Median (mg/L) (SE)	Efficiency (%)	*p*-Value
0	60.3 (35.2)	-	-
5	12.9 (19.4)	78.6	0.07
40	15.1 (16.4)	75.0	0.14
90	0	100.0	<0.05
180	0	100.0	<0.05
300	0	100.0	<0.05
600	0	100.0	<0.05

**Table 3:** Removal efficiency of entecavir by *Microcystis novacekii* in the period of 4 to 30 days of exposure.

Exposure (mg/L)	% Efficiency (EP)				
	Period (Days)				
	4	7	14	21	30
1.2	17.1 (0.9)	17.8 (0.4)	17.8 (0.2)	23.0 (0.3)	28.9 (0.3)
12	13.6 (0.1)	18.1 (10.2)	12.5 (2.6)	19.2 (0.4) *	24.5 (0.6) *
24	13.7 (6.4)	16.8 (27.0)	16.1 (2.1) *	22.5 (0.8) *	22.8 (1.2) *
60	15.4 (1.6)	16.0 (2.0)	16.2 (6.7)	26.7 (33.6)	28.0 (1.2)
120	14.4 (4.0)	14.4 (2.5)	16.6 (3.6)	48.7 (70.0)	21.5 (9.0)

SE: Standard error; * Statistically significant differences in relation to the initial concentration of entecavir (*p* < 0.05).

## Data Availability

The raw data supporting the conclusions of this article will be made available by the authors on request.

## Supplementary Materials

The following supporting information can be downloaded at: https://www.mdpi.com/article/10.3390/toxics12120885/s1. Table 1. Inhibition of *M. novacekii* under exposure to different entecavir concentrations for 72 hours expressed by the inhibition rate (standard deviation). Figure 1. Tukey’s post hoc analysis for *M. novacekii* cellular activity at different entecavir concentrations over 336 hours of exposure.

## References

[1] Shaw T., Locarnini S. (2004). Entecavir for the treatment of chronic hepatitis B. Expert Rev. Anti-Infect. Ther..

[2] Yamanaka G., Wilson T., Innaimo S., Bisacchi G.S., Egli P., Rinehart J.K., Zahler R., Colonno R.J. (1999). Metabolic studies on BMS-200475, a new antiviral compound active against hepatitis B virus. Antimicrob. Agents Chemother..

[3] WHO (2024). Global Hepatitis Report 2024: Action for Access in Low- and Middle-Income Countries.

[4] Brasil (2023). Viral Hepatitis Epidemiological Bulletin.

[5] Sims K.A., Woodland A.M. (2006). Entecavir: A new nucleoside analog for the treatment of chronic hepatitis B infection. Pharmacotherapy.

[6] NCBI (2022). PubChem Compound Summary for CID 135398508, Entecavir. National Center for Biotechnology Information. https://pubchem.ncbi.nlm.nih.gov/compound/Entecavir.

[7] Yao L., Dou W.Y., Ma Y.F., Liu Y.S. (2021). Development and validation of sensitive methods for simultaneous determination of 9 antiviral drugs in different various environmental matrices by UPLC-MS/MS. Chemosphere.

[8] Akkari A.C.S., Munhoz I.P., Tomioka J., Dos Santos N.M.B.F., Dos Santos R.F. (2016). Pharmaceutical innovation: Differences between Europe, USA and ‘pharmerging’ countries. Gest. Prod..

[9] Kornis G.E.M., Braga M.H., de Paula P.A.B. (2014). Recent transformations in the pharmaceutical industry: An examination of the global and Brazilian experience in the 21st century. Physis.

[10] Rashed M.N. (2022). Persistent Organic Pollutants (POPs).

[11] Jain S., Kumar P., Vyas R.K., Pandit P., Dalai A.K. (2013). Occurrence and removal of antiviral drugs in environment: A review. Water Air Soil Pollut..

[12] Ncube S., Madikizela L.M., Chimuka L., Nindi M.M. (2018). Environmental fate and ecotoxicological effects of antiretrovirals: A current global status and future perspectives. Water Res..

[13] Ghernaout D., Elboughdiri N., Ghareba S. (2020). Fenton Technology for Wastewater Treatment: Dares and Trends. Open Access Libr. J..

[14] Vorontsov A.V. (2019). Advancing Fenton and photo-Fenton water treatment through the catalyst design. J. Hazard. Mater..

[15] Parsons S. (2005). Advanced Oxidation Processes for Water and Wastewater Treatment.

[16] Messele S.A., Bengoa C., Stüber F.E., Giralt J., Fortuny A., Fabregat A., Font J. (2019). Enhanced Degradation of Phenol by a Fenton-Like System (Fe/EDTA/H_2_O_2_) at Circumneutral pH. Catalysts.

[17] Kazimierowicz J., Dębowski M., Zieliński M. (2023). Effect of Pharmaceutical Sludge Pre-Treatment with Fenton/Fenton-like Reagents on Toxicity and Anaerobic Digestion Efficiency. Int. J. Environ. Res. Public Health.

[18] Monsalvo V.M., Lopez J., Munoz M., de Pedro Z.M., Casas J.A., Mohedano A.F., Rodriguez J.J. (2015). Application of Fenton-like oxidation as pre-treatment for carbamazepine biodegradation. Chem. Eng. J..

[19] Touliabah H.E.S., El-Sheekh M.M., Ismail M.M., El-Kassas H. (2022). A Review of Microalgae-and Cyanobacteria-Based Biodegradation of Organic Pollutants. Molecules.

[20] Mustafa S., Nawaz H., Maqbool M., Iqbal M. (2021). Microalgae biosorption, bioaccumulation and biodegradation efficiency for the remediation of wastewater and carbon dioxide mitigation: Prospects, challenges and opportunities. J. Water Process Eng..

[21] Lakhani S., Acharya D., Sakariya R., Sharma D., Patel P., Shah M., Prajapati M. (2022). A comprehensive study of bioremediation for pharmaceutical wastewater treatment. Clean. Chem. Eng..

[22] Ahmad I.Z. (2022). The usage of Cyanobacteria in wastewater treatment: Prospects and limitations. Lett. Appl. Microbiol..

[23] Ummalyma S.B., Pandey A., Sukumaran R.K., Sahoo D. (2017). Bioremediation by Microalgae: Current and Emerging Trends for Effluents Treatments for Value Addition of Waste Streams. Biosynthetic Technology and Environmental Challenges.

[24] Wang Q., Liu W., Li X., Wang R., Zhai J. (2020). Carbamazepine toxicity and its co-metabolic removal by the cyanobacteria Spirulina platensis. Sci. Total Environ..

[25] Pan M., Lyu T., Zhan L., Matamoros V., Angelidaki I., Cooper M., Pan G. (2021). Mitigating antibiotic pollution using cyanobacteria: Removal efficiency, pathways and metabolism. Water Res..

[26] Zhou T., Cao L., Zhang Q., Liu Y., Xiang S., Liu T., Ruan R. (2021). Effect of chlortetracycline on the growth and intracellular components of Spirulina platensis and its biodegradation pathway. J. Hazard. Mater..

[27] Hamed S.M., Hozzein W.N., Selim S., Mohamed H.S., AbdElgawad H. (2021). Dissipation of pyridaphenthion by cyanobacteria: Insights into cellular degradation, detoxification and metabolic regulation. J. Hazard. Mater..

[28] Santos M.J.O., Souza C.O., Marcelino H.R. (2023). Blue technology for a sustainable pharmaceutical industry: Microalgae for bioremediation and pharmaceutical production. Algal Res..

[29] Campos M.M.C., Faria V.H.F., Teodoro T.S., Barbosa F.A.R. (2013). Evaluation of the capacity of the cyanobacterium *Microcystis novacekii* to remove atrazine from a culture medium Evaluation of the capacity of the cyanobacterium *Microcystis novacekii* to remove atrazine from a culture medium. J. Environ. Sci. Health Part B.

[30] Fioravante I.A., Barbosa F.A.R., Augusti R., Magalhães S.M.S. (2010). Removal of methyl parathion by cyanobacteria *Microcystis novacekii* under culture conditions. J. Environ. Monit..

[31] Xiao M., Li M., Reynolds C.S. (2018). Colony formation in the cyanobacterium *Microcystis*. Biol. Rev..

[32] Fioravante I.A., Albergaria B., Teodoro T.S., Magalhães S.M.S., Barbosa F., Augusti R. (2012). Removal of 17α-ethinylestradiol from a sterile WC medium by the cyanobacteria *Microcystis novacekii*. J. Environ. Monitiring.

[33] Silva S.R., Barbosa F.A.R., Mol M.P.G., Magalhães S.M.S. (2019). Toxicity for Aquatic Organisms of Antiretroviral Tenofovir Disoproxil. J. Environ. Prot..

[34] Lin S.H., Lo C.C. (1997). Fenton process for treatment of desizing wastewater. Water Res..

[35] Merz J.H., Waters W.A. (1949). The oxidation of aromatic compounds by means of the free hydroxyl radical. J. Chem. Soc..

[36] Carmichael W.W., Gorham P.R. (1974). An improved method for obtaining axenic clones of planktonic blue-green algae. J. Phycol..

[37] Ritz C., Baty F., Streibig J.C., Gerhard D. (2015). Dose-response analysis using R. PLoS ONE.

[38] Sauder D.C., DeMars C.E. (2019). An Updated Recommendation for Multiple Comparisons. Adv. Methods Pract. Psychol. Sci..

[39] Ghiselli G., Jardim W.F., Litter M.I., Mansilla H.D. (2004). Destruction of EDTA using Fenton and photo-Fenton-like reactions under UV-A irradiation. J. Photochem. Photobiol. Chem..

[40] Hua J., Huang M. (2020). Heterogeneous Fenton-like degradation of EDTA in an aqueous solution with enhanced COD removal under neutral pH. Water Sci. Technol..

[41] Xu R., Huang X., Li H., Su M., Chen D. (2018). Simultaneous Removal of Thallium and EDTA by Fenton Process. IOP Conf. Ser. Earth Environ. Sci..

[42] Mackuľak T., Mosný M., Grabic R., Golovko O., Koba O., Birošová L. (2015). Fenton-like reaction: A possible way to efficiently remove illicit drugs and pharmaceuticals from wastewater. Environ. Toxicol. Pharmacol..

[43] Giri A.S., Golder A.K. (2014). Fenton, photo-fenton, H_2_O_2_ Photolysis, and TiO_2_ Photocatalysis for Dipyrone Oxidation: Drug Removal, Mineralization, Biodegradability, and Degradation Mechanism. Ind. Eng. Chem. Res..

[44] Hu Y., Li Y., He J., Liu T., Zhang K., Huang X., Kong L., Liu J. (2018). EDTA-Fe (III) Fenton-like oxidation for the degradation of malachite green. J. Environ. Manag..

[45] United-Nations (2023). Globally Harmonized System of Classification and Labelling of Chemicals (GHS).

[46] Duo L., Yin L., Zhang C., Zhao S. (2019). Chemosphere Ecotoxicological responses of the earthworm Eisenia fetida to EDTA addition under turfgrass growing conditions. Chemosphere.

[47] Oviedo C., Rodríguez J. (2003). EDTA: The chelating agent under environmental scrutiny. Quim. Nova.

[48] Pascual G., Sano D., Sakamaki T., Nishimura O. (2020). Effects of chemical interaction of nutrients and EDTA on metals toxicity to *Pseudokirckneriella subcapitata*. Ecotoxicol. Environ. Saf..

[49] Zhang Y., Zhou M. (2019). A critical review of the application of chelating agents to enable Fenton and Fenton-like reactions at high pH values. J. Hazard. Mater..

[50] Crini G., Lichtfouse E. (2019). Advantages and disadvantages of techniques used for wastewater treatment. Environ. Chem. Lett..

[51] von Sperling M. (2007). Biological Wastewater Treatment Series—Waste Stabilisation Ponds.

[52] Le Van V., Srivastava A., Ko S.R., Ahn C.Y., Oh H.M. (2022). *Microcystis* colony formation: Extracellular polymeric substance, associated microorganisms, and its application. Bioresour. Technol..

[53] Li S., Show P.L., Ngo H.H., Ho S.H. (2022). Algae-mediated antibiotic wastewater treatment: A critical review. Environ. Sci. Ecotechnol..

[54] Dalmora S.L., Sangoi M.S., Nogueira D.R., Silva L.M. (2010). Determination of Entecavir in Tablet Dosage Form. J. AOAC Int..

[55] Malipatil S.M., Athanikar B.S., Dipali M. (2012). Pharma Science Monitor. Pharma Sci. Monit. Int. J. Pharm Sci..

[56] Subashchandrabose S.R., Ramakrishnan B., Megharaj M., Venkateswarlu K., Naidu R. (2013). Mixotrophic cyanobacteria and microalgae as distinctive biological agents for organic pollutant degradation. Environ. Int..

[57] Bai X., Acharya K. (2016). Removal of trimethoprim, sulfamethoxazole, and triclosan by the green alga *Nannochloris* sp.. J. Hazard. Mater..

[58] Xiong Q., Hu L.X., Liu Y.S., Zhao J.L., He L.Y., Ying G.G. (2021). Microalgae-based technology for antibiotics removal: From mechanisms to application of innovational hybrid systems. Environ. Int..

[59] Nie J., Sun Y., Zhou Y., Kumar M., Usman M., Li J., Shao J., Wang L., Tsang D.C.W. (2020). Bioremediation of water containing pesticides by microalgae: Mechanisms, methods, and prospects for future research. Sci. Total Environ..

[60] Silva S., Moreira C., Vasconcelos O., Mol M., Barbosa F., Magalhães S. (2022). Biodegradation of the antiviral tenofovir disoproxyl by a cyanobacteria/bacteria culture. Res. Sq..

[61] Abed R.M.M. (2010). International Biodeterioration & Biodegradation Interaction between cyanobacteria and aerobic heterotrophic bacteria in the degradation of hydrocarbons. Int. Biodeterior. Biodegrad..

[62] Fang Y., Lin G., Liu Y., Zhang J. (2024). Advanced treatment of antibiotic-polluted wastewater by a consortium composed of bacteria and mixed cyanobacteria. Environ. Pollut..

[63] Rempel A., Gutkoski J.P., Nazari M.T., Biolchi G.N., Cavanhi V.A.F., Treichel H., Colla L.M. (2021). Science of the Total Environment Current advances in microalgae-based bioremediation and other technologies for emerging contaminants treatment. Sci. Total Environ..

[64] Zymanczyk-Duda E., Samson S.O., Brzezinska-Rodak M., Klimek-Ochab M. (2022). Versatile Applications of Cyanobacteria in Biotechnology. Microorganisms.

[65] He Q., Wang W., Xu Q., Liu Z., Teng J., Yan H., Liu X. (2022). Microcystins in Water: Detection, Microbial Degradation Strategies, and Mechanisms. Int. J. Environ. Res. Public Health.

