# Supplementary material for: Removal of the Active Pharmaceutical Substance Entecavir from Water via the Fenton Reaction or Action by the Cyanobacterium Microcystis novacekii
Source: Toxics. 2024 Dec 5;12(12):885. doi: 10.3390/toxics12120885 (PMC11679440; doi:10.3390/toxics12120885)

Supplementary material

Microcystis novacekii acute toxicity to entecavir (ETV)

OECD guide 201

Potassium dichromate sensitivity results performed with *M. novacekii* strains had a coefficient of variation (CV) <30% within 30 days with the test condition. ETV did not show acute toxicity in *M. novacekii*. Inhibition rates of all ETV concentrations calculated for 72 (acute) and 336 (chronic) hours of exposure times were negative, indicating cell growth and non-inhibited growth (Table 1). Although with borderline statistical significance, "inhibition" rates were not significantly different over time in different concentrations ($p > 0.05$). As expected, in Tukey's post hoc analysis (Figure 1) under favorable nutrient and light conditions and a pH level between 6.5 and 7.5, significant differences were observed in the number of cells in all concentrations when comparing baseline and the 48-hour and 72-hour periods.

Table S1. Inhibition of *M. novacekii* under exposure to different entecavir concentrations for 72 hours expressed by the inhibition rate (standard deviation).

ETV concentration	Period		p-value
	72 h	336 h	
	I%(SD)	I%(SD)	
**0 mg/L**	N/A	N/A	N/A
**1,2 mg/L**	-17.3 (24.1)	-5.5 (3.8)	0.513
**12 mg/L**	-24.9 (16.7)	-2.5 (4.2)	0.051
**24 mg/L**	-16.2 (18.4)	-2.6 (2.6)	0.275
**60 mg/L**	-24.4 (22.2)	0.6 (3.1)	0.189
**120 mg/L**	-16.5 (22.9)	-2.0 (5.4)	0.388
**180 mg/L**	-11.4 (5.3)	-0.6 (2.6)	0.051
**300 mg/L**	-26.2 (14.8)	-1.2 (3.8)	0.092
**p-value**	0.944	0.602	

Figure S1. Tukey's post hoc analysis for *M. novacekii* cellular activity at different entecavir concentrations over 336 hours of exposure.

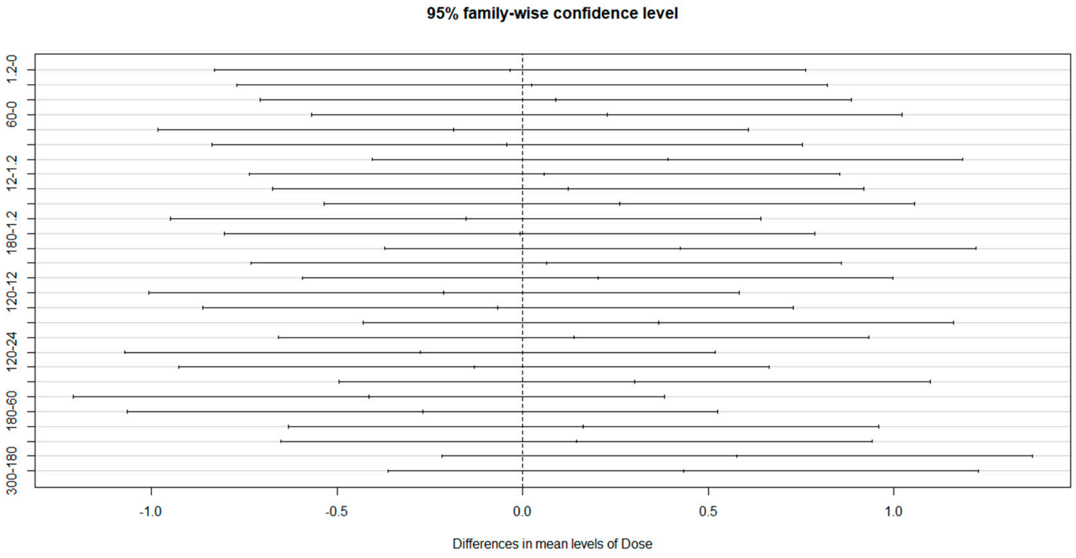

Supplement: Supplementary file 1 [file toxics-12-00885-s001.zip › toxics-3331158-supplementary.pdf]
